# Sex Differences in Pediatric Infectious Diseases

**DOI:** 10.1093/infdis/jiu232

**Published:** 2014-07-15

**Authors:** Maximilian Muenchhoff, Philip J. R. Goulder

**Affiliations:** 1 Department of Paediatrics, University of Oxford,United Kingdom; 2 HIV Pathogenesis Programme, The Doris Duke Medical Research Institute, University of KwaZulu-Natal, Durban, South Africa

**Keywords:** sex, gender, pediatric, infections

## Abstract

The success of the immune response is finely balanced between, on the one hand, the need to engage vigorously with, and clear, certain pathogens; and, on the other, the requirement to minimize immunopathology and autoimmunity. Distinct immune strategies to achieve this balance have evolved in females and males and also in infancy through to adulthood. Sex differences in outcome from a range of infectious diseases can be identified from as early as fetal life, such as in congenital cytomegalovirus infection. The impact of sex hormones on the T-helper 1/T-helper 2 cytokine balance has been proposed to explain the higher severity of most infectious diseases in males. In the minority where greater morbidity and mortality is observed in females, this is hypothesized to arise because of greater immunopathology and/or autoimmunity. However, a number of unexplained exceptions to this rule are described. Studies that have actually measured the sex differences in children in the immune responses to infectious diseases and that would further test these hypotheses, are relatively scarce.

Sex has a major impact on outcome from a range of infectious diseases, starting from the beginning of life. Overall, morbidity and mortality rates are higher in males than in females throughout life [1]. During infancy and childhood, increased susceptibility and severity of infectious diseases for males account for this uneven distribution to a large degree [2]. In humans, females reportedly mount stronger humoral and cellular immune responses to infection or antigenic stimulation than do males [3]. This increased level of immunity can be beneficial in protection against, and clearance of, a proportion of pathogens. However, it can also be detrimental, by increasing immunopathology in certain infectious diseases and by predisposing to autoimmune diseases [4]. The underlying mechanisms for these sexual dimorphisms are multifactorial, including the endocrine and genetic effects on the immune system and physiology, as well as sex-related differences in behavior. From birth to adulthood, the dynamics of sexual maturation overlap with the continuing development of the immune system, showing differential outcomes between the sexes across the age groups after pathogen exposure. In this review, we first delineate the differences in immunity during childhood and between the sexes. In the second part of the review, we further investigate sex-related differences in selected pediatric infections.

## Age- and Sex-Related Differences in Immunity

Increased susceptibility to infections during infancy has been associated with quantitative and functional differences in the specific immune responses generated and a lack of preexisting immunological memory in newborns compared with adults [5]. After birth, the immune system is challenged by a new environment and exposed to a large variety of pathogenic and nonpathogenic antigens after the sheltered existence in utero. There is a fine line between immune responses as a necessary defense mechanism against infectious agents and responses to nonpathogenic agents such as the colonizing intestinal and skin flora that could cause severe immunopathology. The neonatal immune system is finely adapted to reach this balance between immunotolerance and immunoreactivity and shows functional and quantitative differences in the humoral and cellular arms of the immune system compared with that in older children and adults. In the past, neonates were often described as immunodeficient, but more recent research has highlighted that newborns are able to mount effective immune responses that are comparable to those in adults under certain conditions. However, an overall shift toward immunological tolerance in early life is observed across innate and adaptive immunity.

In neonates, CD4^+^ T-helper responses are skewed toward anti-inflammatory T-helper (Th) 2 and regulatory T-cell responses. Effective primary and memory Th1 responses can also be mounted but at a lower magnitude than in adults under most conditions. Antigen specific regulatory T cells induce immunological tolerance, dampen CD4^+^ Th1 responses, and are present at higher levels in infancy. Because CD4^+^ T-helper cells play a pivotal role in orchestrating the responses of many other immune cells, the Th1/Th2 bias in early childhood influences many other immune cell populations and favors responses against extracellular pathogens. Even though CD8^+^ T-cell responses have been observed as early as in fetal life against a variety of pathogens such as cytomegalovirus (CMV), human immunodeficiency virus (HIV), and hepatitis B virus, lower frequencies and functional deficits of cytotoxic T lymphocyte function than in adults have been observed [5]. Dendritic cells, which have a critical antigen presenting role for both CD4^+^ and CD8^+^ T cells, are decreased and show distinct qualitative profiles in children compared with adults [6]. Substantial differences between children and adults have also been found in B-cell immunity, with children showing lower levels, reduced affinity and diversity of T-cell–dependent antibody responses compared with adults, although effective B-cell responses can be induced under certain conditions, as clearly demonstrated by the administration of effective vaccines in infancy [7]. Innate immunity in newborns has adapted to the challenging situation after birth, with a bias toward protection against extracellular bacteria rather than intracellular pathogens [8]. Over time the immune system matures and increasingly shows adult features, with increased proinflammatory and effective memory responses resulting in higher levels of protection against intracellular pathogens.

However, increasing age and progression to adulthood should not be conflated with an increasingly effective immune response. To the contrary, a so-called honeymoon period of childhood infectious diseases has been recognized [9], in which morbidity and mortality from a range of infections, including tuberculosis, influenza, poliomyelitis, mumps, measles, chickenpox, and Epstein-Barr virus infection, follow a U-shaped curve, with maximum disease rates in the first 1–2 years, increasing again only in later childhood, adolescence, and adulthood. Minimal disease in the age groups with the maximum incidence of these infections is consistent with host-pathogen coadaptation [10].

With age, not only does the immune system mature but sex differences also become more apparent. Although full sexual maturation is only reached after puberty, there are fundamental genetic and endocrine differences between the sexes that can shape differential immunity early in life [11]. Long before the peak in sex hormones in adolescence, there is an intermittent surge of sex steroids during infancy, often referred to as *minipuberty*. Besides their role in reproduction and sexual differentiation, sex hormones also influence immune responses [12]. In particular, testosterone, progesterone, and estradiol modulate the functions of lymphocytes, dendritic cells and macrophages, by binding to specific receptors and subsequently binding to hormone response elements in promotor regions. For example, testosterone has been shown to have overall immunosuppressive effects reducing interferon (IFN) γ and interleukin 4 secretion in T cells [13] whereas estrogen can enhance Th1 cellular immune responses at lower doses and increase Th2 responses and humoral immunity at higher concentrations [14]. Differential levels of sex hormones throughout life can therefore influence the appearance of the sexual dimorphism observed in the pathogenesis of infectious diseases. Indeed, for many pathogens, the gap between the sexes in susceptibility and severity of infection is increased during the first year of life, coinciding with the intermittent surge of sex hormones, and then again in puberty, corresponding to the age of sexual maturation and highest variations in the levels of sex hormones between the sexes.

## SEX DIFFERENCES IN OUTCOME FROM SELECTED INFECTIONS IN CHILDHOOD

### Viral Infections

Many of the classic pediatric exanthems, including measles, rubella, erythema infectiosum (“slapped cheek”; parvovirus), and roseola infantum (human herpesvirus 6 and 7), are caused by viruses. With exceptions, the severity and prevalence of viral infections is generally higher in males (Table 1), whereas immunopathology can be increased in females owing to more vigorous innate and adaptive immune responses [34].

**Table 1.** Sex Differences in Viral, Bacterial, and Parasitic Infections in Different age Groups^a^

Pathogen/Infection	Depending Measure Reference	Age, y	Sex Bias
Viral infections			
RSV	Incidence [15]	0–2	M>F
	Severity [15]	0–2	M>F
CMV	Severity [16]	Congenital	M<F
	Incidence [16]	Congenital	M=F
Adenovirus	Incidence [17]	0–14	M>F
	Incidence [17]	>14	M<F
Coxsackievirus	Incidence [17]	0–14	M>F
	Incidence [17]	>14	M<F
HHV-6, HHV-7	Incidence	All	M<F
Measles	Protective immunity [18]	All	M<F
	Incidence [19]	All	M>F
	Severity [20]	All	M<F
	Mortality [20]	All	M<F
	Complications [21, 22]	All	M>F
Mumps	Incidence [17]	0–14	M>F
	Protective immunity [23]	All	M<F
	Complications [24]	All	M>F
VZV, chickenpox	Incidence [25]	0–14	M=F
VZV, chickenpox	Incidence [25]	15–24	M<F
VZV, shingles	Incidence [25]	>14	M<F
Parvovirus B19	Incidence [17]	>2	M<F
HIV	CD4^+^ T-cell count [26, 27]	All	M<F
	Viral load [27]	All	M<F
Bacterial infections			
Tuberculosis	Incidence [28]	0–1, ≥20	M>F
	Severity	0–1, ≥20	M>F
Group A streptococcal pharyngitis	Incidence [29]	All	M>F
	Complications [30, 31]	All	M>F
Leptospirosis	Incidence [28]	<1	M>>F
	Incidence [28]	1–14	M>F
	Incidence [28]	>14	M>>F
Botulism	Incidence	All	M>F
Brucellosis	Incidence	All	M>F
Meningococcal disease	Incidence [28]	0–4	M>F
Pneumococcal disease	Incidence [29]	All	M>F
*Haemophilus Influenzae*	Incidence	0–4	M>F
Pertussis	Incidence [32, 33]	All	M<F
Lower respiratory tract infections	Incidence [29]	All	M>F
	Severity [29]	All	M>F
Mycoplasma pneumoniae	Incidence [17]	All	M<F
Parasitic infections			
Leishmaniasis (cutaneous and visceral)	Incidence [28]	<1	M>>F
	Incidence [28]	1–14	M>F
	Incidence [28]	>14	M>>F
Trypanosomiasis	Incidence	All	M>F
Chagas disease	Incidence	All	M>F
Schistosomiasis	Incidence [28]	All	M>F
Lymphatic filariasis	Incidence	All	M>F
Onchocerciasis	Incidence	All	M>F

Abbreviations: CMV, cytomegalovirus; F, female; HHV, human herpesvirus; HIV, human immunodeficiency virus; M, male; RSV, respiratory syncytial virus; VZV, varicella-zoster virus.

^a^ Information has been extracted from epidemiological surveillance data published by the World Health Organization, the Centers for Disease Control and Prevention, the European Centre for Disease Prevention and Control, the Japanese Infectious Disease Surveillance Center, and specific references as indicated.

Congenital CMV infection is the most significant viral cause of birth defects in industrialized countries. In some affected infants, this can lead to inclusion disease and brain damage, resulting in neurocognitive deficits, sensorineural deafness, and psychomotor retardation. Although congenital infection rates are similar for both sexes, the risk of severe congenital CMV disease leading to brain abnormalities and impaired neurodevelopment is twice as high in females as in males [16]. Damage to the central nervous system does not seem to be mainly caused by CMV infection directly, because studies have revealed a relatively permissive noncytopathic infection of neurons and astrocytes; the damage is rather thought to be induced by immune-inflammatory responses [35]. A study on congenitally CMV-infected fetuses showed that the extent of infiltrating activated cytotoxic CD8^+^ T cells in infected brain tissue is correlated with cerebral damage [36]. Terminally differentiated CD4^+^ and CD8^+^ CMV-specific T cells have also been shown to directly damage endothelial cells, which could increase immune-mediated disease [37]. A comparison of CMV-specific CD4^+^ immune responses in adults between the sexes found a Th1 bias, with higher secretion levels of IFN-γ and interleukin 2 in females [38]. Although sex-specific data on CMV responses in newborns is lacking at present, more vigorous cellular immune responses could explain the increased immunopathology in girls in this entity.

Measles is a childhood infection that has become nearly eradicated in most developed world countries by the availability of an effective vaccine but still causes considerable disease in the developing world. On vaccination, as with most vaccines, females show higher measles antibody titers that persist longer [18]. Interestingly, older studies showed increased mortality rates in females with the use of a high titer vaccine that was consequently discontinued [39]. Although vaccination rates are equal for both sexes [40], the incidence of measles is higher in boys [19]. On infection, however, higher mortality rates have been observed worldwide across all ages for females, with an increasing sex bias in persons of reproductive age, which may be related to higher exposure doses, differences in access to health care, or differential immunity in women [20]. Increased levels of sex hormones in those of reproductive age could also enhance differences in immunological responses, increasing the observed sex bias for this age group. However, in both children and adults, males are more likely to be affected by subacute sclerosing panencephalitis (SSPE) as a complication of measles infection [21]; females show longer latency periods and milder SSPE symptoms [22]. Clearance of measles virus infection is mainly mediated by Th1 responses [41], which are generally stronger in females, but to date no sex-specific data are available for the T-cell responses generated in measles infection.

Recent outbreaks of mumps have shown a higher overall incidence in males, with most patients having had only a single vaccination or none. As with measles vaccination, females show higher and more persistent antibody titers and may therefore be less susceptible to infection [23]. Epididymo-orchitis occurs as a complication of infection in about 15%–30% of men with mumps, whereas oophoritis develops in only 5% of infected postpubertal female patients. Less typical complications of mumps, such as meningitis and encephalitis, also occur more frequently in men [24]. As with the data for measles, relating these sex-specific differences in disease outcome to distinct immune responses is problematic in the absence of relevant studies. In addition, at least some of the described differences (eg, receipt of fewer mumps immunizations in males) seem linked to differences in behavior between or toward boys and girls [42].

Respiratory syncytial virus (RSV) causes one of the most common pathogenic childhood infections; approximately 80% of children experience ≥1 episode by age 1 year. Primary infection is thought to be almost always symptomatic, usually presenting as a mild upper respiratory tract infection and involving the lower respiratory tract only in more severe cases, mainly manifesting as bronchiolitis [15]. Overall incidence rates are higher in boys than in girls, and male sex is a risk factor for severe disease requiring hospital admission [43]. In acute RSV infection early inflammation is mediated by the innate immune system through macrophages and natural killer cells infiltrating the respiratory epithelium. For viral clearance, both CD4^+^ and CD8^+^ T-cell responses are required, although increased T-cell responses have also been associated with increased disease [44], as strikingly illustrated by the disease enhancement caused by the formalin-inactivated vaccine F1-RSV in the 1960s. After vaccination, 80% of vaccinees required hospitalization compared with 5% of controls, and 2 deaths occurred in the former group [45]. Depletion of both T-cell subtypes leads to persistent viral replication but decreased clinical disease [46]. Interestingly, 1 study has shown elevated inflammation markers in acute RSV bronchiolitis in girls in association with better overall outcome, possibly indicating the effect of stronger proinflammatory responses in females [47]. This illustrates the complexity and fine line between an immune response that eliminates virus with minimal disease and one that increases disease. In addition to differential immunity, other factors could also contribute to the observed sex differences. For example, prepubertal boys have shorter airways than age-matched girls, which could increase their susceptibility to lower respiratory tract infections [48].

Croup, or laryngotracheobronchitis, is the most common cause of airway obstruction in children and is typically caused by parainfluenza virus infection after a striking seasonal pattern. The age group most affected is 6 months to 6 years. Higher incidence and hospitalization rates have been consistently observed in males [25]. More severe disease is clearly related to sex differences in the respiratory tract anatomy, as described for RSV disease, as well as to differences in antiviral immunity.

The incidence of chickenpox is similar for the sexes during childhood and adolescence. However, a higher proportion of cases occur in females than in males during the reproductive years. This may be due to greater exposure of women to potentially infected children in childcare settings. Furthermore, reactivation of varicella-zoster virus (VZV) as shingles occurs more often in women, possibly indicating lower levels of immunity in females. Effective memory T-cell responses are thought to maintain VZV latency by preventing viral reactivation [49]. Indeed, in a recent study in healthy adults, VZV-specific CD4^+^ and CD8^+^ memory T-cell responses were detected at higher frequencies in healthy men than in healthy women [26].

HIV infection shows well-documented sex-specific differences in adults as reviewed in a separate article part of this supplement [insert reference here]. However, the higher CD4^+^ T-cell counts observed in females are not confined to infected adults. Large studies of HIV-infected children have demonstrated significantly higher CD4^+^ T-cell counts in vertically infected female subjects naive to highly active antiretroviral therapy (ART) [27] (Mori et al, unpublished data). In HIV-uninfected children, absolute CD4^+^ T-cell counts and percentages are also significantly higher in girls than in boys (Mori et al, unpublished data) [50], as early as the first day of life. Despite higher CD4^+^ T-cell counts in females, the rate of progression to HIV disease does not differ in children or in adults by sex [29]. One possible concern, therefore, would be that because the criteria for ART initiation are based on absolute CD4^+^ T-cell counts or percentages, girls would be starting ART inappropriately late. However, a large study of >2000 HIV-infected children shows no sex differences in morbidity and mortality after ART (Mori et al, unpublished data).

### Bacterial Infections

In children, bacterial infections mainly involve the respiratory tract. Across all age groups, upper respiratory tract infections, such as sinusitis and tonsillitis, are found more frequently in females, whereas lower respiratory tract infections are more common and more severe in males [51]. However, there are also many exceptions to this simplified pattern. For group A streptococcal pharyngitis*,* for example*,* 1 study found a slightly higher incidence in boys [30], and rheumatic fever as an inflammatory disease after streptococcal infection was also observed at higher rates in boys than in girls [31, 32]. A recent reappearance of pertussis has been observed in Europe, with higher incidence rates in females and most severe cases occurring in children before they reach vaccination age [33], and another study from the United States also found higher mortality rates for infected females [17]. Finally, *Mycoplasma pneumoniae*, the main pathogen of atypical community-acquired pneumonia that is often found in adolescents and young adults, shows a clear predominance in females, from studies in Japan [52]. However, no data currently available provide a biological concept for these observations.

### Vaccine-Mediated Effects

The current World Health Organization program on immunization is estimated to prevent more than 2 million childhood deaths each year [53]. However, these vaccines also seem to have effects beyond the intended reduction of disease caused by the relevant pathogen. For example, vaccination against both bacille Calmette-Guérin (BCG) and measles reduces mortality due to causes other than tuberculosis and measles, respectively [54, 55]. It is proposed that this may be due to a shift in the Th1/Th2 cytokine balance, which is in favor of Th2 in infancy and especially so in males, as described in the earlier section Age- and Sex-Related Differences in Immunity. It is also proposed that the measles vaccine reduces the nonmeasles mortality, especially in girls [56].

BCG reportedly has an epigenetic “training” effect on innate immunity in healthy human volunteers, leading to increased monocyte production of IFN-γ, tumor necrosis factor, and interleukin 1b, in response to unrelated bacterial and fungal pathogens [57]. This effect was mediated through epigenetic regulation via histone-3 lysine-4 trimethylation of the *NOD2* gene, an intracellular pattern recognition receptor that recognizes muramyl dipeptide, which has been linked with increased expression of proinflammatory cytokines, such as interleukin 12 [58]. In BCG-vaccinated mice, 100% were protected against disseminated candidiasis, compared with only 30% of controls [57].

The nonspecific effects of the diphtheria-tetanus-pertussis vaccine, in contrast to those of the live vaccines, are to increase mortality after administration in girls [59]. Again, this is hypothesized to be the result of an altered balance between the Th1 and Th2 cytokines, but studies of transcriptome changes observed in girls after diphtheria-tetanus-pertussis vaccination had inconclusive findings [28].

### Parasitic Infections

Disparities in the incidence and severity of infections with parasites between sexes have been observed and studied intensively. The sex differences in parasitic infection in adults are reviewed in a separate article as part of this supplement [insert reference]. Interestingly, sex differences in some parasitic diseases show a striking age pattern, as illustrated by the example of cutaneous leishmaniasis. Its incidence shows a significant male bias during infancy that decreases during early and late childhood [60]. With onset of puberty, the ratio of male to female case patients starts to increase again, with a peak in the reproductive years and a subsequent decline in the elderly. Hence, the sex bias seems to be increased at the stages of life characterized by high levels of sex hormones, at “minipuberty” in infancy and at puberty and early adulthood. Protective immunity against and resolution of cutaneous leishmaniasis is mediated predominantly by Th1 responses, whereas Th2 responses have been associated with susceptibility and disease exacerbation [61]. Because testosterone promotes production of Th2 cytokines and estrogen enhances proinflammatory Th1 responses [62], differential levels of sex hormones may contribute to this sexual dimorphism in disease outcome.

## CONCLUSIONS

It is clear that a strong sexual dimorphism can be observed for susceptibility and disease outcome in many childhood infections, long before sexual maturation and full expression of sexual traits. Sex biases in some infectious conditions vary with age, indicating the influence of differential levels of sex hormones throughout different stages of life. In the first years after birth, the increased risk of infection and the balance between immunotolerance and immunoreactivity is a delicate state of the immune system that might be even more susceptible to minor differences between the sexes than later in adult life. For many pathogens susceptibility is higher in males, which can be partially explained by the observation of stronger Th1 immune responses in females, but higher levels of proinflammatory immunity also predispose females to increased immunopathology in some infections. This simplified generalization, however, does not apply to all infectious conditions, reflecting a more complex interplay between sex and immunity, in addition to the differences in behavior between and toward boys and girls, which require further research.

## References

[1] Lozano R, Naghavi M, Foreman K (2012). Global and regional mortality from 235 causes of death for 20 age groups in 1990 and 2010: a systematic analysis for the Global Burden of Disease Study 2010. Lancet.

[2] Anker M (2007). Addressing sex and gender in epidemic-prone infectious diseases.

[3] Fish EN (2008). The X-files in immunity: sex-based differences predispose immune responses. Nat Rev Immunol.

[4] Amur S, Parekh A, Mummaneni P (2012). Sex differences and genomics in autoimmune diseases. J Autoimmun.

[5] Prendergast AJ, Klenerman P, Goulder PJ (2012). The impact of differential antiviral immunity in children and adults. Nat Rev Immunol.

[6] Kollmann TR, Crabtree J, Rein-Weston A (2009). Neonatal innate TLR-mediated responses are distinct from those of adults. J Immunol.

[7] Siegrist CA (2007). The challenges of vaccine responses in early life: selected examples. J Comp Pathol.

[8] Levy O (2007). Innate immunity of the newborn: basic mechanisms and clinical correlates. Nat Rev Immunol.

[9] Ahmed R, Oldstone MB, Palese P (2007). Protective immunity and susceptibility to infectious diseases: lessons from the 1918 influenza pandemic. Nat Immunol.

[10] Anderson RM, May RM, Anderson B (1991). Infectious diseases of humans: dynamics and control.

[11] Ober C, Loisel DA, Gilad Y (2008). Sex-specific genetic architecture of human disease. Nat Rev Genet.

[12] Bouman A, Heineman MJ, Faas MM (2005). Sex hormones and the immune response in humans. Hum Reprod Update.

[13] Araneo BA, Dowell T, Diegel M, Daynes RA (1991). Dihydrotestosterone exerts a depressive influence on the production of interleukin-4 (IL-4), IL-5, and gamma-interferon, but not IL-2 by activated murine T cells. Blood.

[14] Straub RH (2007). The complex role of estrogens in inflammation. Endocr Rev.

[15] Nair H, Nokes DJ, Gessner BD (2010). Global burden of acute lower respiratory infections due to respiratory syncytial virus in young children: a systematic review and meta-analysis. Lancet.

[16] Picone O, Costa JM, Dejean A, Ville Y (2005). Is fetal gender a risk factor for severe congenital cytomegalovirus infection?. Prenat Diagn.

[17] Haberling DL, Holman RC, Paddock CD, Murphy TV (2009). Infant and maternal risk factors for pertussis-related infant mortality in the United States, 1999 to 2004. Pediatr Infect Dis J.

[18] Mossong J, O'Callaghan CJ, Ratnam S (2000). Modelling antibody response to measles vaccine and subsequent waning of immunity in a low exposure population. Vaccine.

[19] Green MS (1992). The male predominance in the incidence of infectious diseases in children: a postulated explanation for disparities in the literature. Int J Epidemiol.

[20] Garenne M (1994). Sex differences in measles mortality: a world review. Int J Epidemiol.

[21] Gutierrez J, Issacson RS, Koppel BS (2010). Subacute sclerosing panencephalitis: an update. Dev Med Child Neurol.

[22] Manayani DJ, Abraham M, Gnanamuthu C, Solomon T, Alexander M, Sridharan G (2002). SSPE—the continuing challenge: a study based on serological evidence from a tertiary care centre in India. Indian J Med Microbiol.

[23] Dominguez A, Plans P, Costa J (2006). Seroprevalence of measles, rubella, and mumps antibodies in Catalonia, Spain: results of a cross-sectional study. Eur J Clin Microbiol Infect Dis.

[24] Hviid A, Rubin S, Muhlemann K (2008). Mumps. Lancet.

[25] Segal AO, Crighton EJ, Moineddin R, Mamdani M, Upshur RE (2005). Croup hospitalizations in Ontario: a 14-year time-series analysis. Pediatrics.

[26] Klein NP, Holmes TH, Sharp MA (2006). Variability and gender differences in memory T cell immunity to varicella-zoster virus in healthy adults. Vaccine.

[27] Ruel TD, Zanoni BC, Ssewanyana I (2011). Sex differences in HIV RNA level and CD4 cell percentage during childhood. Clin Infect Dis.

[28] Orntoft NW, Thorsen K, Benn CS (2013). Leukocyte transcript alterations in West-African girls following a booster vaccination with diphtheria-tetanus-pertussis vaccine. Scand J Clin Lab Invest.

[29] Marinda E, Humphrey JH, Iliff PJ (2007). Child mortality according to maternal and infant HIV status in Zimbabwe. Pediatr Infect Dis J.

[30] Lin MH, Chang PF, Fong WK, Yen CW, Hung KL, Lin SJ (2003). Epidemiological and clinical features of group A *Streptococcus* pharyngitis in children. Acta Paediatr Taiwan.

[31] Breda L, Miulli E, Marzetti V, Chiarelli F, Marcovecchio ML (2013). Rheumatic fever: a disease still to be kept in mind. Rheumatology (Oxford).

[32] Ravisha MS, Tullu MS, Kamat JR (2003). Rheumatic fever and rheumatic heart disease: clinical profile of 550 cases in India. Arch Med Res.

[33] Celentano LP, Massari M, Paramatti D, Salmaso S, Tozzi AE (2005). Resurgence of pertussis in Europe. Pediatr Infect Dis J.

[34] Klein SL (2012). Sex influences immune responses to viruses, and efficacy of prophylaxis and treatments for viral diseases. Bioessays.

[35] McCarthy M, Auger D, Whittemore SR (2000). Human cytomegalovirus causes productive infection and neuronal injury in differentiating fetal human central nervous system neuroepithelial precursor cells. J Hum Virol.

[36] Gabrielli L, Bonasoni MP, Santini D (2012). Congenital cytomegalovirus infection: patterns of fetal brain damage. Clin Microbiol Infect.

[37] van de Berg PJ, Yong SL, Remmerswaal EB, van Lier RA, ten Berge IJ (2012). Cytomegalovirus-induced effector T cells cause endothelial cell damage. Clin Vaccine Immunol.

[38] Villacres MC, Longmate J, Auge C, Diamond DJ (2004). Predominant type 1 CMV-specific memory T-helper response in humans: evidence for gender differences in cytokine secretion. Hum Immunol.

[39] Knudsen KM, Aaby P, Whittle H (1996). Child mortality following standard, medium or high titre measles immunization in West Africa. Int J Epidemiol.

[40] de Melker H, Pebody RG, Edmunds WJ (2001). The seroepidemiology of measles in Western Europe. Epidemiol Infect.

[41] Griffin DE, Ward BJ, Esolen LM (1994). Pathogenesis of measles virus infection: an hypothesis for altered immune responses. J Infect Dis.

[42] Hill K, Upchurch DM (1995). Gender differences in child health: evidence from the demographic and health surveys. Population Dev Rev.

[43] Borchers AT, Chang C, Gershwin ME, Gershwin LJ (2013). Respiratory syncytial virus—a comprehensive review. Clin Rev Allergy Immunol.

[44] Openshaw PJ, Tregoning JS (2005). Immune responses and disease enhancement during respiratory syncytial virus infection. Clin Microbiol Rev.

[45] Kim HW, Canchola JG, Brandt CD, et al (1969). Respiratory syncytial virus disease in infants despite prior administration of antigenic inactivated vaccine. Am J Epidemiol.

[46] Graham BS, Bunton LA, Wright PF, Karzon DT (1991). Role of T lymphocyte subsets in the pathogenesis of primary infection and rechallenge with respiratory syncytial virus in mice. J Clin Invest.

[47] Nagayama Y, Tsubaki T, Nakayama S (2006). Gender analysis in acute bronchiolitis due to respiratory syncytial virus. Pediatr Allergy Immunol.

[48] Ronen O, Malhotra A, Pillar G (2007). Influence of gender and age on upper-airway length during development. Pediatrics.

[49] Fleming DM, Cross KW, Cobb WA, Chapman RS (2004). Gender difference in the incidence of shingles. Epidemiol Infect.

[50] Bunders M, Cortina-Borja M, Newell ML (2005). Age-related standards for total lymphocyte, CD4+ and CD8+ T cell counts in children born in Europe. Pediatr Infect Dis J.

[51] Falagas ME, Mourtzoukou EG, Vardakas KZ (2007). Sex differences in the incidence and severity of respiratory tract infections. Respir Med.

[52] Eshima N, Tokumaru O, Hara S (2012). Age-specific sex-related differences in infections: a statistical analysis of national surveillance data in Japan. PloS One.

[53] Pollard AJ, Green C, Sadarangani M, Snape MD (2013). Adolescents need a booster of serogroup C meningococcal vaccine to protect them and maintain population control of the disease. Arch Dis Child.

[54] Shann F (2010). The non-specific effects of vaccines. Arch Dis Child.

[55] Aaby P, Roth A, Ravn H (2011). Randomized trial of BCG vaccination at birth to low-birth-weight children: beneficial nonspecific effects in the neonatal period?. J Infect Dis.

[56] Aaby P, Martins CL, Garly ML (2010). Non-specific effects of standard measles vaccine at 4.5 and 9 months of age on childhood mortality: randomised controlled trial. BMJ.

[57] Kleinnijenhuis J, Quintin J, Preijers F (2012). Bacille Calmette-Guerin induces NOD2-dependent nonspecific protection from reinfection via epigenetic reprogramming of monocytes. Proc Natl Acad Sci U S Am.

[58] Wen H, Dou Y, Hogaboam CM, Kunkel SL (2008). Epigenetic regulation of dendritic cell-derived interleukin-12 facilitates immunosuppression after a severe innate immune response. Blood.

[59] Aaby P, Ravn H, Roth A (2012). Early diphtheria-tetanus-pertussis vaccination associated with higher female mortality and no difference in male mortality in a cohort of low birthweight children: an observational study within a randomised trial. Arch Dis Child.

[60] Guerra-Silveira F, Abad-Franch F (2013). Sex bias in infectious disease epidemiology: patterns and processes. PLoS One.

[61] Alexander J, Bryson K (2005). T helper (h)1/Th2 and *Leishmania*: paradox rather than paradigm. Immunol Lett.

[62] Snider H, Lezama-Davila C, Alexander J, Satoskar AR (2009). Sex hormones and modulation of immunity against leishmaniasis. Neuroimmunomodulation.

